# THE ROLE OF SENSE OF COMMUNITY IN CHANGING THE HEALTH-PROMOTING EFFECT OF BUILT ENVIRONMENT: A COMMUNITY SURVEY

**DOI:** 10.1093/geroni/igz038.1031

**Published:** 2019-11-08

**Authors:** Jennifer Y M Tang, Cheryl Chui, Tuen Yi Chiu, Rebecca Chiu, Vivian W Lou, Michael Tse, Angela Y M Leung, Terry Lum

**Affiliations:** 1 Sau Po Centre on Ageing, The University of Hong Kong, Pok Fu Lam, Hong Kong; 2 Department of Social Work and Social Administration, Pok Fu Lam, Hong Kong; 3 Department of Urban Planning and Design, the University of Hong Kong, Pok Fu Lam, Hong Kong; 4 The University of Hong Kong, Hong Kong, P.R.C., Hong Kong; 5 Centre for Sports and Exercise, the University of Hong Kong, Pok Fu Lam, Hong Kong; 6 Centre for Gerontological Nursing, School of Nursing, The Hong Kong Polytechnic University, Hong Kong, Hong Kong

## Abstract

Previous research that studies the impact of built environment on health often attribute the enabling effects of environment on physical activity participation and opportunities for social interaction. Few studies have explored how the role of subjective feeling, such as the feeling of connectedness with the community, affects the association between built environment and physical and mental health. We conducted a cross-sectional survey with 2,247 residents aged 50 years or above in five districts in Hong Kong. We tested the mediation effect of sense of community in the relationship between physical environment and health using the path analysis. We administered a questionnaire to assess the residents’ perceived age-friendliness of outdoor spaces and buildings in the district. We used the Brief Sense of Community Scale and the 12-item Short-form Health Survey to measure sense of community and physical and mental health. We found that age-friendliness of outdoor spaces was modestly correlated with mental health (r = 0.10, P < 0.001) but not with physical health (r = 0.02, P = 0.4), whereas age-friendliness of buildings correlated with both (r = 0.05, P = 0.01; r = 0.06, P = 0.004). Sense of community mediated 25.9% of the total effect between outdoor space and physical health, 20.4% between outdoor space and mental health, and 42.5% between service and building on physical health. To conclude, sense of community was a partial mediator of the environment-health relationship. Future design of built environment should take into consideration its potential influence on sense of community and health.

